# Depression, Anxiety and Quality of Life in Patients with Atopic Dermatitis and Psoriasis

**DOI:** 10.3390/medicina62010164

**Published:** 2026-01-14

**Authors:** Arina Arnīte, Vanda Bondare-Ansberga, Lelde Reinberga, Ilona Hartmane, Ingmārs Mikažāns

**Affiliations:** 1 Department of Dermatology and Venerology, Riga Stradins University, Baznicas Street 18, LV-1010 Riga, Latvia; 2 Riga 1st Hospital, Bruninieku Street 5, LV-1001 Riga, Latvia

**Keywords:** atopic, DLQI, eczema, GAD-7, PHQ-9, psoriasis

## Abstract

*Background and Objectives*: Atopic dermatitis and psoriasis are life-long inflammatory diseases affecting more than just the skin. Although their link with mental comorbidities has been established, the role of using self-assessment questionnaires is still debated. The aim of our study was to evaluate differences in the quality of life (DLQI) as well as depression (PHQ-9) and anxiety (GAD-7) questionnaire data and determine their link with individual patient and skin disease factors. *Materials and Methods*: Demographic, clinical and questionnaire data were collected from Riga 1st hospitals archive. For statistical evaluation, the Mann–Whitney U test and Spearman’s rank correlation coefficient were used. *Results*: The median DLQI for atopic dermatitis and psoriasis was 10.5 and 10, respectively. The prevalence among women with atopic dermatitis who had a PHQ-9 ≥ 10 was 42.9%, compared to 50.0% in men, and GAD-7 ≥ 10 prevalence was 14.3% and 20.0%, respectively. Psoriatic women had a PHQ-9 ≥ 10 prevalence of 25.0% compared with 28.9% in men. The prevalence of GAD-7 ≥ 10 was 20.0% in females and 15.8% in males. GAD-7 score was elevated in patients with psoriatic genital involvement. Multiple positive correlations were noted between PHQ-9, GAD-7 and DLQI scores. *Conclusions*: Patient quality of life and prevalence of anxiety and depression symptoms are impacted by psoriasis and atopic dermatitis, with similar patterns observed across genders and comorbidities. Genital involvement could be associated with more severe anxiety symptoms. The correlations between PHQ-9, GAD-7 and DLQI scores indicate that further evaluation might be necessary if quality of life is impaired.

## 1. Introduction

Inflammatory skin diseases are widespread, with previous studies showing psoriasis prevalence of more than 60 million people globally and atopic dermatitis of around 101 million adults [1,2]. They are chronic and affect the organism beyond the skin, often causing a mental burden [3].

Atopic dermatitis is a chronic inflammatory disease of the skin that mostly affects children; however, it can persist into or manifest de novo during adulthood [4]. Its acute form is characterized by erythema, papules and vesicles with exudate, and its chronic form is marked by signs of xerosis, lichenification and dyspigmentation. Patients often have pruritus, which results in excoriations [5]. Atopic dermatitis has a well-established association with food and airborne allergies, bronchial asthma, rhinitis and conjunctivitis [6,7,8]. Furthermore, individuals with atopic dermatitis face issues such as disturbed relationships due to rejection and stigma, changes in sleep patterns and disruptions in leisure and physical activities, all of which can lower quality of life [9].

Psoriasis is a skin disease which is more common in North America and some of the Western and Northern European countries [1,10,11]. Clinically, it is characterized by infiltrated papulosquamous plaques with a white silvery scale located in virtually any location; however, other (for example, inverse, guttate or palmoplantar) psoriasis types can also be seen [12]. Patients often have different comorbidities related to continuous inflammatory processes in the body, such as metabolic syndrome and atherosclerosis, that can promote myocardial infarction and ischemic stroke [13,14]. These comorbidities, together with skin presentation and symptoms like pruritus and pain, can impact the quality of life and social functioning because of discrimination and lowered self-esteem [15]. Thus, anxiety and depression are quite commonly encountered in this population group [16]. And all of these factors together over a period of time can lead to cumulative life-course impairment, posing a risk of not living life to its fullest potential [17].

Moreover, prior research has also demonstrated that acknowledging the psychiatric comorbidities and receiving care can improve patients’ well-being, and has therapeutic efficacy for their skin diseases as well [18,19,20].

Whilst mental health impairment has a well-established association with atopic dermatitis and psoriasis, data specifically examining the link with gender and body area involvement remain limited. Thus, the aim of our study was to analyze differences in the quality of life, as well as depression and anxiety questionnaire data, and measure their relationships with individual patient and skin disease factors.

## 2. Materials and Methods

### 2.1. Characteristics of Subjects

**Figure 1:**
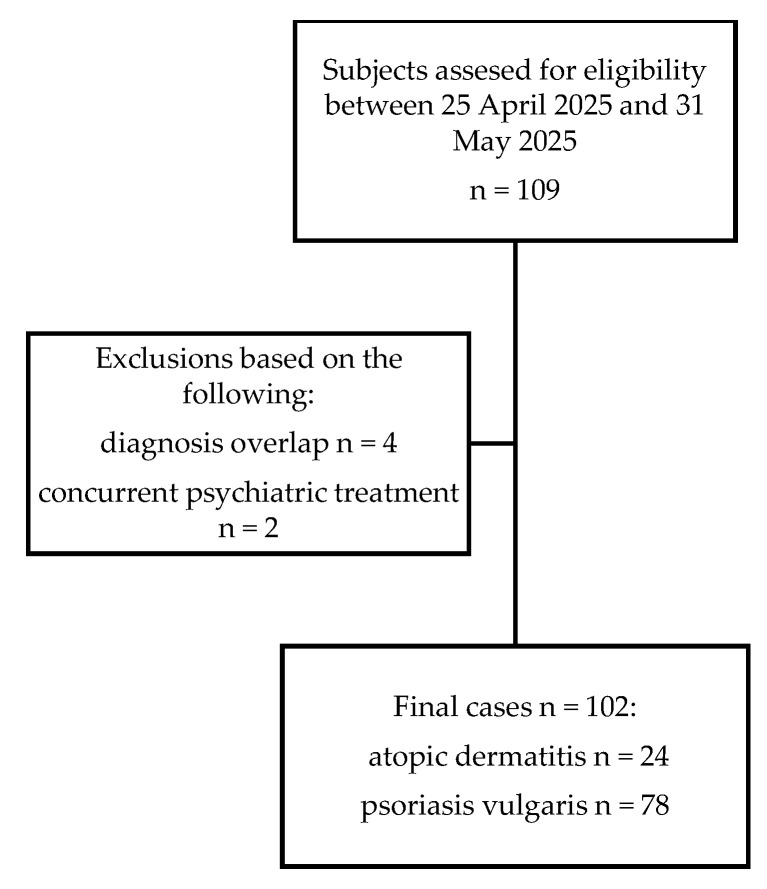
Flow diagram for the retrospective study selection.

Our retrospective research was conducted in accordance with the Helsinki Declaration and approved by the ethical committee at Riga Stradins University on the 7 April 2025 (Project Nr. 2-PEK-4/614/2025), and by the ethical committee at Riga 1st Hospital on the 25 April 2025 (Protocol Nr. 05/2025). Patient informed consent was waived due to the retrospective and observational study design, and the decision was based on article 10, paragraph 8 of the Law on Patients’ Rights of the Republic of Latvia. The patient demographic and disease data, as well as psychiatric questionnaire data, were obtained from Riga 1st Hospital’s archive. A total of 102 patient samples were obtained. Inclusion criteria were being at least 18 years of age and having a diagnosis of psoriasis or atopic dermatitis made by a dermatologist. Exclusion criteria were overlap of atopic dermatitis and psoriasis, other inflammatory dermatologic conditions and current psychiatric treatment (Figure 1).

### 2.2. Measures

In our study, multiple scales were employed to assess clinical data. Body surface area (BSA) shows the percentage of body affected. A BSA of <3% indicates a mild, 3–10% moderate and >10% severe involvement [21]. The Psoriasis Area Severity Index (PASI) was also used, which measures the extent of psoriasis using the percentage of body region involved and the severity of erythema, induration and scaling [22]. The eczema area and severity index (EASI), which is used to evaluate atopic dermatitis, considers body part involvement and assesses erythema, induration, excoriation and lichenification [23].

Health-related quality of life was assessed using the dermatology life quality index (DLQI), which is validated for adult use. The DLQI is an instrument that evaluates several aspects of daily living with 10 questions and has a total score from 0 to 30. A result closer to 30 indicates a greater influence of disease on quality of life [24].

The patient health questionnaire (PHQ-9) is a tool used to evaluate the presence of depressive symptoms based on the Diagnostic and Statistical Manual of Mental Disorders, Fourth Edition (DSM-IV) criteria. A PHQ-9 score of 0–4 points indicate minimal, 5–9 mild, 10–14 moderate, 15–19 moderately severe and 20–27 severe depression symptoms [25]. The questionnaire was adapted into Latvian by Rancans et al. and has been used as a screening tool for depression, especially in primary care [26].

The generalized anxiety disorder scale-7 (GAD-7) is a self-questionnaire that is based on DSM-IV criteria and makes it possible to screen patients for generalized anxiety disorder. It classifies the severity of anxiety in four groups: minimal (0–4), mild (5–9), moderate (10–14) and severe (15–21) [27]. It was validated for the Latvian population by Vrublevska et al. in 2022 in a nationwide study [28].

### 2.3. Statistical Analysis

IBM SPSS software version 29.0 (IBM company, North Castle, Armonk, NY, USA) was used to process data. Descriptive statistics were used to characterize the demographic and disease data of all participants. In this study, categorical variables are described by frequencies (*n*) with percentages and continuous data as median (Med) with first and third quartiles (Q1, Q3). The Mann–Whitney U test was used to examine the differences between studied variables. The *p*-value was <0.05 as the significance level was set at 5%. The Benjamini–Hochberg false discovery rate (FDR (BH)) correction as well as rank–biserial correlation (r_rb) were also applied. To evaluate correlations between patient and disease factors, we used Spearman’s rank correlation coefficient, where R < 0.2 indicated a negligible, R = 0.2–0.4 a weak, R = 0.4–0.6 a moderate, R = 0.6–0.8 a strong and R = 0.8–1 a very strong correlation.

## 3. Results

Among the 102 patients enrolled in the study, 48 (47.1%) were male and 54 (52.9%) were female. Patients were split according to their diagnosis: those with atopic dermatitis (*n* = 24, 23,5%) and those with psoriasis vulgaris (*n* = 78, 76.5%).

Patients with atopic dermatitis had median BSA, EASI and DLQI scores of 9.0 (Q1: 5.0–Q3: 11.0), 10.4 (Q1: 5.2–Q3: 16.7) and 10.5 (Q1: 6.2–Q3: 19.7), respectively, correlating with a moderate disease presentation (Table 1).

In patients with psoriasis vulgaris, the median score for BSA was 9.0 (Q1: 4.9–Q3: 14.2), PASI 9.6 (Q1: 5.0–Q3: 15.0) and DLQI 10.0 (Q1: 5.0–Q3: 15.0), relating to a moderate disease severity (Table 2).

Mental health questionnaire results varied between both disease groups. The median PHQ-9 score for patients with atopic dermatitis patients was 8.0 (Q1: 3.2–Q3: 12.7) and median GAD-7 was 5.0 (Q1: 2.2–Q3: 8.5). The prevalence among women with atopic dermatitis who had a PHQ-9 ≥ 10 was 42.9% (95% CI 14.3–71.4), compared to 50.0% (95% CI 20.0–80.0) in men. GAD-7 ≥ 10 prevalence was 14.3% (95% CI 0.0–35.7) in females and 20.0% (95% CI 0.0–50.0) in males.

Psoriatic patients had a median PHQ-9 of 6.0 (Q1: 3.0–Q3: 10.0) and a median GAD-7 of 4.0 (Q1: 2.0–Q3: 8.0). PHQ-9 ≥ 10 prevalence among women was 25.0% (95% CI 12.5–37.5), compared with 28.9% (95% CI 15.8–44.7) in men. The prevalence of GAD-7 ≥ 10 was 20.0% (95% CI 7.5–32.5) in females and 15.8% (95% CI 5.3–28.9) in males.

There were 12 individuals (50.0%) with atopic dermatitis who had significant comorbidities made by clinician judgement, out of which the most common was bronchial asthma (*n* = 6, 25.0%).

In the psoriasis vulgaris group, there were 34 patients (43.6%) who were without any other disease. However, for those who had clinically significant comorbidities, the most common were primary arterial hypertension (*n* = 30, 38.5%), type 2 diabetes mellitus (*n* = 7, 9.0%), chronic gastritis (*n* = 4, 5.1%), hypothyroidism (*n* = 3, 3.8%), bronchial asthma (*n* = 3, 3.8%) and coronary artery disease (*n* = 2, 2.6%). The only statistically significant difference noted between individuals with comorbidities and those without was related to age in the psoriasis group, where patients who had comorbidities were older (Med 59.5 (Q1: 44.7–Q3: 66.0)) than those who did not (Med 38.5 (Q1: 27.0–Q3: 47.0)) (U = 274.5, *p* = <0.001). Significance remained after applying Benjamini–Hochberg false discovery rate (FDR-BH = 0.01) and rank–biserial correlation (r_rb) was 0.64.

Multiple statistically significant differences between genders were observed among patient groups. In atopic dermatitis, males had a higher BSA than females; however, after the *p*-value correction with the FDR (BH) test, the significance lowered (Table 3).

In patients with psoriasis vulgaris, a statistically significant difference between genders was noted in age, which was higher in females; BSA that was similar to atopic dermatitis patients was higher in males, and PASI had an elevated score, again, in males. But, also in this patient group, the significance lowered after implementing FDR (BH) correction (Table 4).

Moreover, some psoriasis-affected body regions showed a difference in age parameter. Patients with scalp psoriasis were younger (Med 42.0 years (Q1: 30.0–Q3: 61.0)) than those without it (Med 54.0 years (Q1: 41.0–Q3: 63.0)) (U = 546.0, *p* = 0.032, FDR (BH) = 0.01). A similar association was noted in age and facial lesions (U = 205.0, *p* = 0.044, FDR (BH) = 0.01), where patients with this area involvement had a median age of 39.0 years (Q1: 26.5–Q3: 51.0), but those without it had a median age of 52.5 (Q1: 37.2–Q3: 63.7) years. Patients without genital involvement were older (Med 53.0 years (Q1: 37.5–Q3: 63.5)) than those with it (32.0 years (Q1: 24.5–Q3: 41.0)) (U = 135.0, *p* = 0.006). Test significance for this result remained after applying correction (FDR (BH) = 0.01, r_rb = 0.82). And lastly, GAD-7 score was elevated in patients with genital involvement (Med 8.0 (Q1: 5.0–Q3: 11.5)) as opposed to patients with no involvement (Med 4.0 (Q1: 2.0–Q3: 7.0) (U = 184.5, *p* = 0.048). And, although the significance lowered after correction (FDR (BH) = 0.01), patients with genital psoriasis had significantly higher odds of clinically significant anxiety (GAD-7 ≥ 10) in a binary logistic regression (OR 4.72, 95% CI 1.08–20.65, *p* = 0.04). The result remained significant after adjustment for gender (OR = 4.91, 95% CI 1.11–21.81, *p* = 0.04), BSA (OR = 4.73, 95% CI = 1.07–20.79, *p* = 0.04) and PASI (OR = 4.96, 95% CI = 1.11–22.14, *p* = 0.04), but the result was no longer statistically significant when age was included as a covariate (OR = 3.29, CI = 0.681–15.84, *p* = 0.14).

Spearman’s rank correlation coefficient showed a very strong positive correlation in atopic dermatitis between duration of disease and age, as well as between BSA and EASI scores. Strong positive correlation was seen between PHQ-9 and GAD-7 and between DLQI and PHQ-9 and GAD-7. EASI showed a moderate positive correlation with duration of disease and age, as did BSA and DLQI. Moderate negative correlation was noted with duration of disease and PHQ-9 (Table 5).

In psoriasis vulgaris, Spearman’s rank correlation coefficient showed a very strong correlation between PASI and BSA, a moderate correlation between GAD-7 and PHQ-9 and a weak correlation between DLQI and PASI, BSA, GAD-7 and PHQ-9 (Table 6).

## 4. Discussion

**Figure A1:**
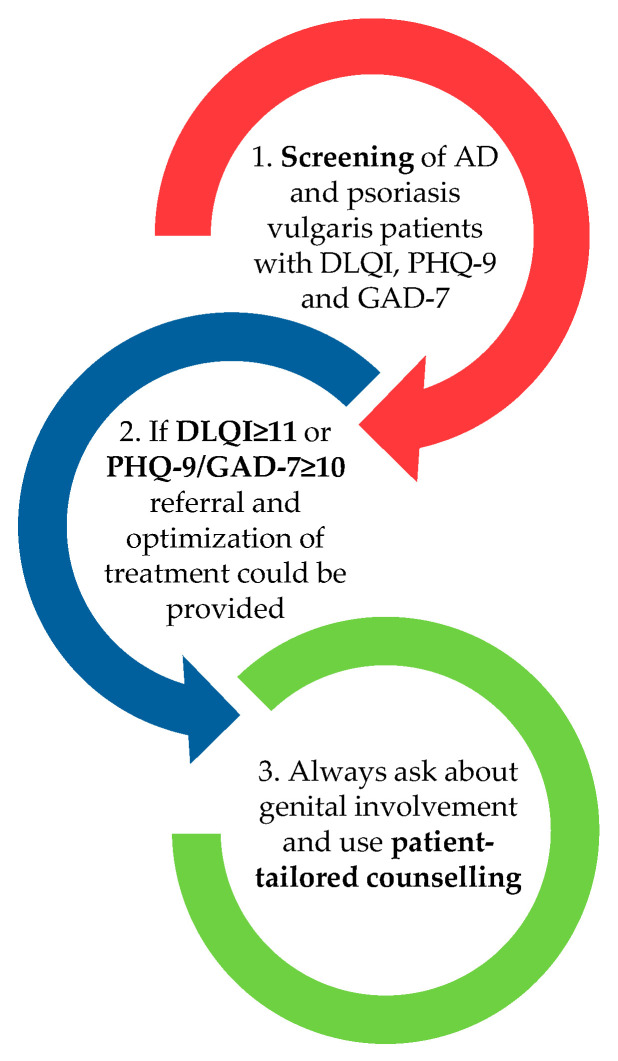
Three step workflow for patients with atopic dermatitis and psoriasis vulgaris.

During the last decade, a special interest has been growing in mental health and its effect on chronic and life-altering diseases, such as heart failure, diabetes mellitus, cancer and chronic kidney disease [29,30,31,32]. Inflammatory skin pathologies extend further than just physical manifestations, with depression and anxiety being more prevalent in atopic dermatitis and psoriasis patients than in the general population [33,34]. The results of our research are similar. In Latvia, the point prevalence of clinically significant depressive symptoms (PHQ-9 ≥ 10) is 4.8% in men and 7.7% in women [35]. And, for generalized anxiety disorder symptoms (GAD-7 ≥ 10), the point prevalence in Latvian men is 2.7% and in women—4.9% [36]. Our study shows that, in atopic dermatitis, PHQ-9 ≥ 10 prevalence was 42.9% in women and 50.0% in men, while GAD-7 ≥ 10 prevalence was 14.3% and 20.0%. In psoriasis vulgaris, women had a PHQ-9 ≥ 10 prevalence of 25.0% and GAD-7 ≥ 10 of 20.0%, versus males, who had respective scale prevalences of 28.9% and 15.8%. It could be explained by patients experiencing unpleasant symptoms, social stigma due to visible skin lesions and impaired daily functioning [9,15]. Furthermore, our patients reported a DLQI score that is linked to mostly moderate to very large disease effects on their life quality [37]. Clinically, PHQ-9/GAD-7 ≥ 10 could serve as a screening tool for further referral of the patient, and DLQI > 10 could imply that evaluation and improvement of therapy might be needed (Appendix A, Figure A1).

Atopic dermatitis is frequently associated with allergic rhinitis and bronchial asthma, and psoriasis with diseases like atherosclerosis, metabolic syndrome, diabetes mellitus and psoriatic arthritis, all of which could influence the mental well-being of patients [6,7,13,14,38,39]. However, in our study, the only statistically significant difference between patients with and without comorbidities was in age, where affected patients were older. We did not observe any difference between DLQI, PAH-9 and GAD-7 results in the aforementioned groups. It might be because atopic dermatitis and psoriasis have a major effect on a patient’s psycho-emotional well-being themselves [9,16]. It has been proven that one of the pathogenetic mechanisms of depression is due to neuroinflammation, which is often linked to obesity or metabolic syndrome. However, the inflammatory patterns are also similar in psoriasis, proving that skin disease is not only having an impact on the relationships and self-worth of the patient, but that it also plays just as important a role in the overall physiology of psychiatric diseases [40,41,42].

Gender is often evaluated in mental comorbidity and skin disease severity, and also in our study certain differences in studied parameters were observed [36,43]. Although after the FDR (BH) correction, the significance of the *p*-value lowered for multiple analyses, these results still could potentially be of clinical significance. In this study, males with psoriasis had overall larger BSA and PASI scores, and psoriatic females were older, which corresponds to other studies [43]. It is also important to note that there could be biological skin differences between genders regarding the sensitivity to stimuli, dermal thickness and pH [44]. In addition, females might be more likely to seek medical care and use topical at-home treatment than males [45,46]. Importantly, even though studies about gender differences and mental disturbances are conflicting, there were no differences observed between anxiety and depression scores between groups in our study, suggesting that both could be potentially affected [47,48,49].

Patients with younger age in our study had scalp, facial and genital psoriasis more often. Similar results have been noted in a study made by Trettel et al., which concluded that younger patients had scalp psoriasis more than older age groups [50]. Although the mechanism is poorly understood, it could be that these patients have an earlier disease onset and more severe presentation [51]. These difficult-to-treat areas have been linked to anxiety and depression before; however, our study provides an additional insight by showing an association between psoriatic genital involvement and elevated GAD-7 scores [52]. However, the small number of participants in this subgroup, limits the statistical power of this result, thus highlighting the need for further research.

Our study also explored multiple subgroup correlations, revealing potential patterns that could have clinical relevance. Firstly, in atopic dermatitis patients, age, duration of disease and EASI positively correlated with each other, which could be because older patients can have a longer duration of the condition and impaired skin barrier function, which impacts disease severity [53]. There was a negative correlation noted between the duration of atopic dermatitis and PHQ-9, which means that depressive symptoms can be more severe earlier in the disease. A study made by Chatrath et al. also concluded that AD symptoms fluctuate over time, especially together with severity [54]. Whilst the underlying reasons are still unclear, other studies have shown that illness perception and coping strategies could have an association with self-rated physical impairment in these patients [55]. Patient education and respective interventions, which can take implementation time, could be associated with lower disease severity [56]. Mental health studies can also be affected by survivorship bias, where patients with a higher mental health burden could be less likely to continue with follow-ups [57]. DLQI correlated with BSA in both groups and PASI in the psoriasis group, which has been noted in other studies; as with increasing disease severity, life quality can decrease [58]. Yet, the correlation between DLQI and BSA/PASI in the psoriasis group was weak, signalling that there could be a discrepancy between patient-perceived and objective disease severity, which aligns with other studies that report weak or variable associations between these scales [59,60]. Thus, it might be best to incorporate both objective and subjective measures in clinical practice while evaluating disease severity [61]. Moreover, in both disease groups, there was a positive correlation noted between BSA and PASI and EASI, which comes as no surprise, as both PASI and EASI take into consideration the affected body area percentage [22,23]. Regarding mental health questionnaires, DLQI correlated with both PHQ-9 and GAD-7 scores, and PHQ-9 and GAD-7 results correlated positively with each other. Other studies have noted similar correlations not only in psoriasis but also in rosacea and vitiligo [62,63,64]. This altogether highlights the value of applying DLQI alongside mental health screening tools.

However, our study has limitations, like a retrospective study design and a relatively small sample size, as well as a disproportion of patients with atopic dermatitis and psoriasis that did not allow us to evaluate all of the factor differences between both diseases. Moreover, the samples came only from patients with studied skin conditions; there was no control group. Due to the fact that patients with ongoing psychiatric treatment were not included in the study to exclude confounding of the results, the true prevalence of anxiety and depression could be underestimated. And finally, because all data were collected from a single inpatient hospital, patients with moderate disease severity could be over-represented, potentially affecting depression and anxiety prevalence estimates. As a result, the findings might not be generalizable to other in- or outpatient clinic settings. Further research, including a larger patient population, especially in the atopic dermatitis group, and patients with existing psychiatric treatment with the use of diagnostic, not only screening, tools, could provide a benefit.

Despite limitations, the key strength of our study was the analysis of actual clinical data from routine dermatology practice, which provides insight into the psychological burden of patients who receive care in clinical settings. In addition, another strength is the use of validated and widely accepted instruments for disease severity (BSA, PASI, EASI, DLQI) and psychological screening (PHQ-9 and GAD-7). Lastly, the combined assessment of atopic dermatitis’s and psoriasis vulgaris’s objective and subjective severity, as well as mental health outcomes, allows a more comprehensive evaluation of patient burden.

A rapidly emerging area of interest is the application of artificial intelligence (AI) in medicine, including dermatology. Before visiting a specialist, patients often consult AI platforms with images and descriptions of their skin lesions. Research has shown that AI systems based on large language models (LLMs) may efficiently assist dermatologic image detection and categorization, highlighting their potential in clinical practice [65,66]. A further clinical application would be to integrate patient-reported scales such as DLQI, PHQ-9 and GAD-7, along with disease severity indexes (BSA, PASI, EASI), into an electronic health record that automatically flags high-risk profiles, generates standardized, non-stigmatizing clinical language and suggests a patient-tailored next-step programme. Such AI-assisted triage could improve screening, reduce missed cases and make questionnaire-based assessment easier and more productive.

## 5. Conclusions

Patients’ quality of life can be impacted by psoriasis and atopic dermatitis, and both conditions are associated with elevated levels of anxiety and depression symptoms, with similar patterns observed across genders and comorbidity status. Genital involvement could possibly have an association with more severe anxiety symptoms. In addition, the correlations between PHQ-9, GAD-7 and DLQI scores indicate that, in the case of impaired quality of life, further evaluation might be necessary.

## Figures and Tables

**Table 1:** Baseline and disease characteristics of patients with atopic dermatitis.

Parameter	Value
Age, Med (Q1–Q3)	31.0 (25.5–46.2)
Gender, *n* (%)	
Females	14 (58.3)
Males	10 (41.7)
Duration of the disease, Med (Q1–Q3)	27.5 (21.2–44.2)
Body part affected, *n* (%):	
Scalp	1 (4.2)
Face	10 (41.7)
Body	24 (100.0)
Palms	1 (4.2)
Genitals	1 (4.2)
EASI, *n* (%):	
1.1–7.0 (mild)	7 (29.2)
7.1–21.0 (moderate)	15 (62.5)
21.1–50.0 (severe)	2 (8.3)
DLQI, *n* (%)	
No effect on patient’s life (0–1)	1 (4.2)
Small effect on patient’s life (2–5)	4 (16.7)
Moderate effect on patient’s life (6–10)	7 (29.2)
Very large effect on patient’s life (11–20)	8 (33.3)
Extremely large effect on patient’s life (21–30)	4 (16.7)

Abbreviations: Med—median; Q1—first quartile; Q3—third quartile; *n*—count; EASI—Eczema area and severity index; DLQI—Dermatology life quality index.

**Table 2:** Baseline and disease characteristics of patients with psoriasis vulgaris.

Parameter	Value
Age, Med (Q1–Q3)	50.5 (35.0–62.0)
Gender, *n* (%):	
Females	40 (51.3)
Males	38 (48.7)
Duration of the disease, Med (Q1–Q3)	12.5 (5.0–27.0)
Body part affected, *n* (%):	
Scalp	39 (50.0)
Face	10 (12.8)
Body	67 (85.9)
Genitals	9 (11.5)
Palms	12 (15.4)
Soles	6 (7.7)
Nails	10 (12.8)
PASI, *n* (%):	
<5 (mild)	17 (21.8)
5–10 (moderate)	34 (43.6)
>10 (severe)	27 (34.6)
DLQI, *n* (%):	
No effect on patient’s life (0–1)	5 (6.4)
Small effect on patient’s life (2–5)	17 (21.8)
Moderate effect on patient’s life (6–10)	18 (23.1)
Very large effect on patient’s life (11–20)	32 (41.0)
Extremely large effect on patient’s life (21–30)	6 (7.7)

Abbreviations: Med—median; Q1—first quartile; Q3—third quartile; *n*—count; PASI—Psoriasis area and severity index; DLQI—Dermatology life quality index.

**Table 3:** Comparison of atopic dermatitis patient and disease characteristics by gender.

Parameter	Females (*n* = 14, 58.3%)	Males (*n* = 10, 41.7%)	U	*p*-Value	FDR (BH)	r_rb
BSA, Med (Q1–Q3)	6.0 (4.0–10.2)	10.0 (7.5–27.0)	104.5	0.042	0.01	−0.49
EASI, Med (Q1–Q3)	9.0 (3.7–12.8)	14.5 (8.7–20.6)	103.0	0.056	0.01	−0.47
GAD-7, Med (Q1–Q3)	4.0 (2.5–7.5)	5.5 (1.7–10.2)	79.0	0.625	0.02	−0.13
Age, Med (Q1–Q3)	32.5 (26.5–48.2)	31.0 (22.2–44.7)	63.5	0.709	0.03	0.09
DLQI, Med (Q1–Q3)	11.5 (4.7–19.2)	10.5 (7.0–20.2)	75.0	0.796	0.04	−0.07
PHQ-9, Med (Q1–Q3)	7.0 (3.0–12.5)	9.0 (3.5–14.0)	72.5	0.886	0.04	−0.04
Duration of atopic dermatitis, Med (Q1–Q3)	27.0 (21.7–46.0)	28.5 (8.2–42.7)	68.5	0.931	0.05	0.02

Abbreviations: *n*—count; Q1—first quartile; Q3—third quartile; BSA—body surface area; EASI—Eczema area and severity index; DLQI—Dermatology life quality index; PHQ-9—Patient health questionnaire-9; GAD-7—Generalized anxiety disorder scale-7, U—Mann–Whitney U statistic; FDR (BH)—Benjamini–Hochberg false discovery rate; r_rb—rank–biserial correlation.

**Table 4:** Comparison of psoriasis vulgaris patient and disease characteristics by gender.

Parameter	Females (*n* = 40 (51.3%))	Males (*n* = 38 (48.7%))	U	*p*-Value	FDR (BH)	r_rb
Age, Med (Q1–Q3)	58.0 (39.5–64.7)	42.5 (29.7–58.5)	514.0	0.014	0.01	0.32
PASI, Med (Q1–Q3)	6.9 (3.2–10.9)	10.0 (7.6–17.1)	985.0	0.024	0.01	−0.30
BSA, Med (Q1–Q3)	5.0 (3.0–10.0)	10.0 (7.0–17.2)	973.5	0.032	0.02	−0.28
Duration of psoriasis in years, Med (Q1–Q3)	15.0 (5.0–38.5)	10.0 (4.7–18.5)	608.5	0.129	0.03	0.20
PHQ-9, Med (Q1–Q3)	5.0 (2.2–10.5)	7.0 (3.0–10.0)	859.0	0.321	0.04	−0.13
GAD-7, Med (Q1–Q3)	4.0 (2.0–8.0)	5.0 (2.7–7.2)	816.0	0.574	0.04	−0.07
DLQI, Med (Q1–Q3)	10.5 (5.0–15.0)	10.0 (4.7–15.2)	755.5	0.964	0.05	0.01

Abbreviations: *n*—count; Q1—first quartile; Q3—third quartile; BSA—body surface area; PASI—Psoriasis area and severity index; DLQI—Dermatology life quality index; PHQ-9—Patient health questionnaire-9; GAD-7—Generalized anxiety disorder scale-7; U—Mann–Whitney U statistic; FDR (BH)—Benjamini–Hochberg false discovery rate; r_rb—rank–biserial correlation.

**Table 5:** Spearman’s rank correlation coefficient revealing correlations between factors in atopic dermatitis.

Factor 1	Factor 2	R	*p*-Value
Very strong correlation (R = 0.80–1.00)			
Duration of disease	Age	0.910	<0.001
BSA	EASI	0.907	<0.001
Strong correlation (R = 0.60–0.79)			
PHQ-9	GAD-7	0.753	<0.001
DLQI	PHQ-9	0.734	<0.001
DLQI	GAD-7	0.638	<0.001
Moderate correlation (R = 0.40–0.59)			
Duration of disease	EASI	0.488	0.016
Age	EASI	0.485	0.016
BSA	DLQI	0.429	0.037
Duration of disease	PHQ-9	−0.474	0.019

Abbreviations: R—Spearman’s rank correlation coefficient; BSA—body surface area; EASI—Psoriasis area and severity index; DLQI—Dermatology life quality index; PHQ-9—Patient health questionnaire-9; GAD-7—Generalized anxiety disorder scale-7.

**Table 6:** Spearman’s rank correlation coefficient revealing correlations between different factors in psoriasis vulgaris.

Factor 1	Factor 2	R	*p*-Value
Very strong correlation (R = 0.80–1.00)			
PASI	BSA	0.967	<0.001
Moderate correlation (R = 0.40–0.59)			
GAD-7	PHQ-9	0.593	<0.001
Weak correlation (R = 0.2–0.39)			
DLQI	PASI	0.399	<0.001
DLQI	BSA	0.386	<0.001
DLQI	GAD-7	0.343	0.002
DLQI	PHQ-9	0.315	0.005

Abbreviations: R—Spearman’s rank correlation coefficient; BSA—body surface area; PASI—Psoriasis area and severity index; DLQI—Dermatology life quality index; PHQ-9—Patient health questionnaire-9; GAD-7—Generalized anxiety disorder scale-7.

## Data Availability

The original contributions presented in this study are included in the article. Further inquiries can be directed to the corresponding author.

## Abbreviations

The following abbreviations are used in this manuscript:

BSA	Body Surface Area
PASI	Psoriasis Area Severity Index
EASI	Eczema Area and Severity Index
DLQI	Dermatology Life Quality Index
PHQ-9	Patient Health Questionnaire-9
DSM-IV	Diagnostic and Statistical Manual of Mental Disorders, Fourth Edition
GAD-7	Generalized Anxiety Disorder Scale-7
Med	Median
Q1	First Quartile
Q3	Third Quartile
*n*	Count
R	Spearman’s Rank Correlation Coefficient
U	Mann–Whitney U Statistic
FDR (BH)	Benjamini–Hochberg False Discovery Rate
p_pb	Rank–Biserial Correlation
LLM	Large Language Model
